# 1183. Implementation of a Nursing and Certified Nursing Assistant Antimicrobial Stewardship Competency-Based Educational Module at a Community Health System

**DOI:** 10.1093/ofid/ofad500.1023

**Published:** 2023-11-27

**Authors:** Elisabeth Chandler, Ashley L Cubillos, Stephanie Ducas, Iraida Reyes, Janet Stedcke, Mary B Saunders

**Affiliations:** Gulf Coast Medical Center, Fort Myers, Florida; Banner North Colorado Medical Center, Loveland, Colorado; NCH Healthcare System, Naples, Florida; Tifton Regional Medical Center, Lenox, Georgia; Lee Health, Fort Myers, Florida; Lee Health, Fort Myers, Florida

## Abstract

**Background:**

The 2019 edition of the CDC Core Elements of Hospital Antibiotic Stewardship Programs created a new category of nursing-based interventions to optimize antimicrobial therapy, however many institutions have not yet implemented these actions. Additionally, accrediting bodies such as the Joint Commission are implementing requirements for multidisciplinary competency-based training as a part of updated standards. Nurses and certified nursing assistants (CNAs) play a valuable role in antimicrobial stewardship (AMS). However, awareness and education pertaining to AMS for nurses and CNAs is not consistent across practice settings, nor do standard recommendations for AMS competency-based education exist. This project aimed to implement and evaluate AMS competency-based education for nursing and CNA staff at a large community health system.

**Methods:**

Implementation of a nursing and CNA AMS online learning module was a multi-step initiative within a community health system. A stepwise approach included assessment of key knowledge gaps, development of educational content specific to the user, creation of an engaging module that included patient cases and knowledge assessment questions, and modification of content to fit within an online learning system. A description of the initiative after competency implementation was assessed.

**Results:**

The online learning module was implemented in December 2022, and the analysis was performed in March 2023. A total of 1,671 nurses and 591 CNAs completed the education in the 3-month time frame. The median duration for competency completion was 26 minutes for nurses (expected duration 30 minutes) and 13 minutes for CNAs (expected duration 20 minutes). Though a passing score of 80% was required for competency completion, 48% of nurses and 74% of CNAs received a passing score of 100%.

**Conclusion:**

Utilizing a standardized online learning system to disseminate an AMS module to nursing and CNA staff achieved a rapid dissemination of AMS competency-based education, enhancing system compliance with CDC recommendations and accrediting organization requirements. Future investigation is needed regarding the impact of educational initiatives on AMS practices for nursing and CNA staff.

**Disclosures:**

**All Authors**: No reported disclosures

